# Screening of potential chemical marker with interspecific differences in *Pterocarpus* wood and a spatially-resolved approach to visualize the distribution of the characteristic markers

**DOI:** 10.3389/fpls.2023.1133848

**Published:** 2023-02-14

**Authors:** Bo Liu, Qian Chen, Lina Tang, Liming Zhu, Xianwu Zou, Botao Li, Wei Fan, Yuejin Fu, Yun Lu

**Affiliations:** ^1^ Research Institute of Wood Industry, Chinese Academy of Forestry, Beijing, China; ^2^ State Key Laboratory of Tree Genetics and Breeding, Chinese Academy of Forestry, Beijing, China

**Keywords:** *Pterocarpus santalinus* and *Pterocarpus tinctorius*, MALDI-TOF-MSI, chemical fingerprint, species-level timber identification, wood anatomy

## Abstract

Profiling the spatial distributions and tissue changes of characteristic compounds with interspecific differences is critical to elucidate the complex species identification during tree species traceability, wood anti-counterfeiting verification and timber trade control. In this research, in order to visualize the spatial position of characteristic compounds in two species with similar morphology (*Pterocarpus santalinus* and *Pterocarpus tinctorius*), a high coverage MALDI-TOF-MS imaging method was used to found the mass spectra fingerprints of different wood species. 2-Mercaptobenzothiazole matrix was used to spray wood tissue section to enhance the detection effect of metabolic molecules, and the mass spectrometry imaging data were obtained. Based on this technology, the spatial location of fifteen potential chemical markers with remarkable interspecific differences in 2 *Pterocarpus* timber species were successfully obtained. Distinct chemical signatures obtained from this method can promote rapid identification at the wood species level. Thus, matrix-assisted laser desorption/time-of-flight/ionization mass spectrometry imaging (MALDI-TOF-MSI) provides a spatial-resolved way for traditional wood morphological classification and breaking through the limitations of traditional wood identification technology.

## Introduction

The strong demand for wood products in the market has led to the rapid growth of the global timber trade volume year on year. After a decline in the early 2000s, the trade volume of illegally harvested timber rose again (Tacconi, 2012). In recent years, tropical timber trade control represented by the species listed in the Convention on International Trade in Endangered Species of Wild Fauna and Flora (CITES) Appendix has been a hot and sensitive issue of global concern. Interpol estimates that 15% - 30% of the global timber trade violates the laws or international treaties of the host country(Irwin, 2019). At present, there were more than 500 wood species belonging to 20 families, 31 genera, have been listed in the CITES Appendix (CITES, 2022), and gradually extended its control scope to various tropical wood species. CITES strives to strengthen coordination and cooperation with other relevant international organizations to further strengthen the management of international timber trade (Belton et al., 1998).

**Figure 1:**
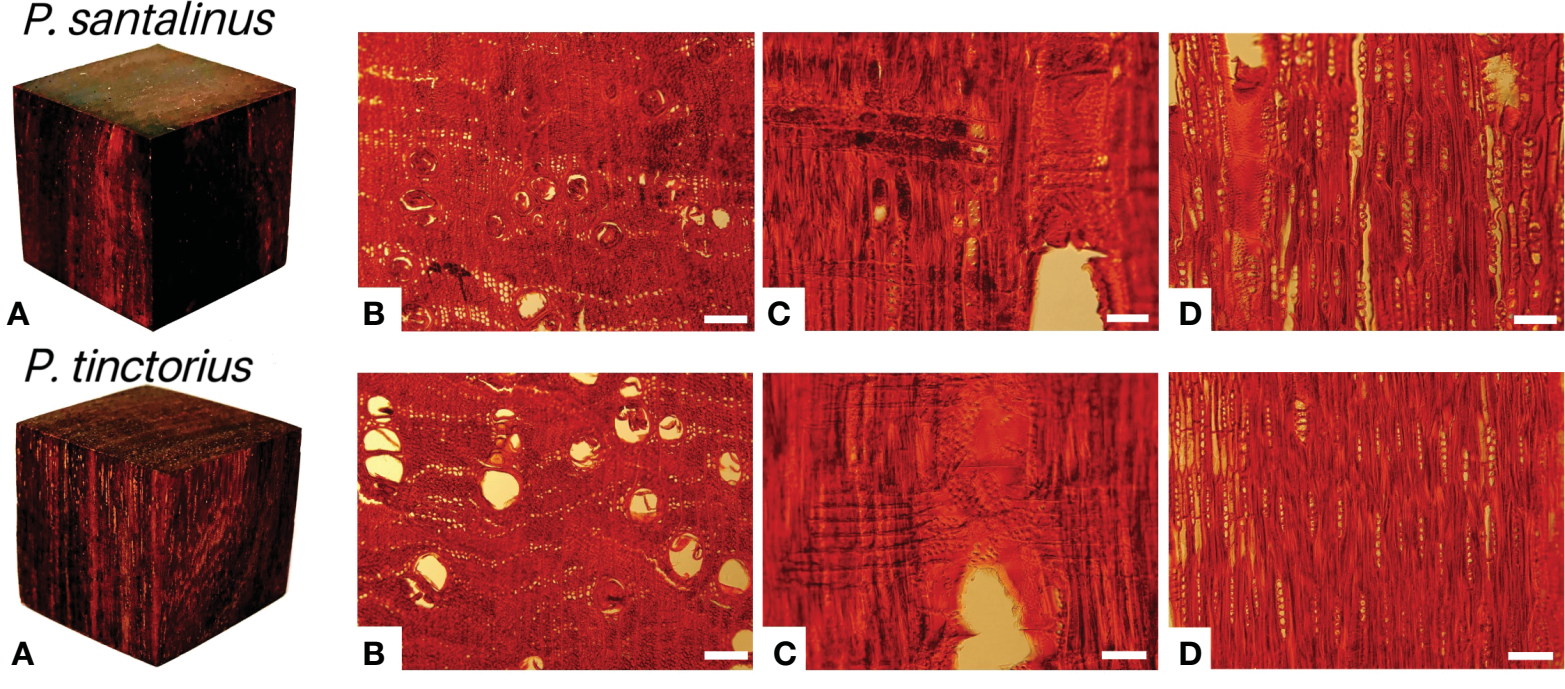
Macroscopic **(A)** and microscopic photographs **(B–D)** of *P. santalinus* and *P. tinctorius*. **(B–D)**-Transverse, radial and tangential sections of *P. santalinus* and *P. tinctorius*, respectively. Scale bars, 200 μm **(B)** and 100 μm **(C, D)**.

*Pterocarpus santalinus* (*P. santalinus*) and *Pterocarpus tinctorius* (*P. tinctorius*) have been listed in CITES Appendix II for control and protection. Their logs, wood chips, powders and extracts were prohibited from import, export and trade without an export license or reexport certificate (The World Conservation Union (IUCN), 2022). The macrostructural and microstructural differences between *P. santalinus* and *P. tinctorius* were not obvious (**Figure 1**). Therefore, it is difficult to distinguish the species differences between them by using the traditional identification of wood anatomy based on structural characteristics of wood. These two species were misidentified frequently both in customs inspections and market consumption. But they were quite different in terms of value and trade restrictions. The traditional timber genus identification based on wood anatomical features has been widely used in botany. However, it is still very difficult to distinguish wood at the species level according to its morphology alone, which has certain limitations (Ng et al., 2016; Liu et al., 2018; Jiao et al., 2019).

In view of the fact that wood morphological methods can only distinguish wood, with the development of analytical instruments and technology, molecular biological analysis and chemical metabolite analysis have been developed for wood identification (Jiao et al., 2018a). Although wood DNA barcode technology can identify ‘species’, it is expensive, DNA extraction is difficult, usually involves complex procedures, and the technology is not popular. Moreover, for the wood species identification, it still lacks the optimization and determination of universal DNA barcode (Yu et al., 2017; Jiao et al., 2018b). Stable isotope technology can realize the identification of ‘species’, and can also track the origin of wood. However, this method requires high sample sources and has low resolution (generally greater than 250km). In the field of plant classification and recognition, a general standard database has not been formed yet (Jiao et al., 2014). Some macroscopic features of wood, such as wood color and texture, come from the chemical composition inside the wood (Irwin, 2019). Therefore, the difference of wood histochemical characteristics is also the external manifestation of genomic differences among different species. Each species can leave specific metabolomic evidence under different growth conditions such as different development stages, environment, soil and climate and so on (Gudi et al., 2015). Therefore, wood species identification by using wood metabonomic characteristics combined with various mass spectrometry has become one of the efforts of wood researchers in recent years.

As for chemical analysis, because of its high sensitivity and selectivity, GC-MS (Gas chromatography - mass spectrometry) and LC-MS (Liquid chromatography - mass spectrometry) have been widely employed in life analysis, plant metabolomics, environmental detection, food evaluation and other research fields (Ashton and Rabi, 2016). The mobile phase of GC-MS is gas (such as helium), which is very suitable for analyzing low-grade and low-boiling metabolites that are easy to vaporize, and for samples of thermally stable molecules. The mobile phase of LC-MS is liquid, which is not affected by sample volatility and thermal stability. However, sample treatment is required before determination, such as uniform grinding or cutting, and extraction of samples. Compared with GC-MS, LC-MS has more longer sample pretreatment and higher cost of organic solvent consumption. In addition, although the sample preparation process of DART-MS (Direct analysis in real time mass spectrometry) technology is simple (Zhang et al., 2019a), but like the other two technologies, the location and spatial concentration information of the target in the tissue cannot be obtained, which is very useful for studying the correlation between wood chemical composition and wood macro- and micro-structural characteristics in wood identification.

MALDI-TOF-MSI (Matrix-assisted laser desorption/ionization time-of-flight mass spectrometry imaging) is and effective technique to characterize tissue molecule profiles, which has the advantages of accuracy, rapidity and high throughput, especially in the aspect of molecular specificity (Lu et al., 2017). As a label free detection technology, it can analyze various types of compounds, such as metabolite of small molecules, lipids, peptides, glycans, proteins and drug molecules, and can be used to find disease markers, explore differences between different groups, or determine the spatial distribution of target molecules. Compared with traditional methods, such as GC-MS and LC-MS, MALDI-TOF-MSI does not require complex sample pretreatment, labeling and chromatographic separation, thus can reduce the change and loss of active components, which is more suitable for the analysis of complex samples (Cornett et al., 2007). MALDI-TOF-MSI allows the spatially resolved detection of thousands of compounds in a single tissue section, and has been widely used in many biological systems of plants (Debois et al., 2014; Shroff et al., 2015) For instance, MALDI-TOF-MSI was used for the analysis of the functional metabolite of ginseng, the active ingredient of aloe, the development regulation of *Arabidopsis thaliana*, the ingredients of ginkgo leaves, and the flavonoids in Huangceng, etc. (Kuo et al., 2019; Sun et al., 2019; Liu et al., 2019a; Li et al., 2020; Sun et al., 2021). The localization of metabolites in roots, leaves and tender stems was visualized by MALDI-TOF-MSI for the study of the classification of ginsenosides and the distribution of flavonoids and other metabolites. Therefore, MALDI-TOF-MSI has great potential in visualizing the structural components of wood tissue. In this study, the MALDI-TOF-MSI approach was applied for the rapid identification of two precious *Pterocarpus* species controlled by CITES, and the spatial location of the characteristic compounds with interspecific differences in the two species was imaged for the first time.

## Materials and methods

### Materials and reagents

Wood specimens of *P. santalinus* and *P. tinctorius* heartwood were supplied from Wood Collections of Chinese Academy of Forestry (WOODPEDIA). Indium-tin oxide (ITO) coated glass slides and double-sided conductive tape were purchased from China’s Sinopharm. ACN in chromatographic grade were purchased from Merck (Darmstadt, Germany). TFA were purchased from Sigma-Aldrich (Taufkirchen, Germany). ddH_2_O was purified by filtration system (Sartorius arium-comfort II, Germany). 2-MBT were supplied by Bruker Beijing laboratory (Bruker, Germany).

### Matrix coating

Matrix coating was carried out with an ImagePrep electronic matrix sprayer (Bruker Daltonics, Germany). The spray of matrix was performed according to the spray method provided by the instrument. The MALDI matrix for each wood section detection was 6 mL 2-MBT, which was prepared by ACN (12mg/mL), TFA and double distilled water (80:0.2:19.8, v/v/v). In previous researches, 2-MBT, DMCA, 2,5-dihydroxybenzoic acid, α- cyano-4-hydroxycnmic acid, graphene oxide, and silver nanoparticles have been used as matrices. It was found that 2-MBT had better ionization efficiency, more effective signal peaks and better signal intensity for wood slices (He et al., 2019; Liu et al., 2019b).

### Sample preparation

Small wood blocks of about 1 cm^3^ were cut from each specimen, without any chemical or boiling treatment. Transverse sections of 20 µm in thickness were cut with a sliding microtome (Leica SM2010R, Germany). All sections were held flatly by slides for optical imaging and matrix coating. Before entering the mass spectrometer, the wood slices were sticked on indium-tin oxide (ITO) coated glass slides with double-sided conductive tape by using clean tweezers. 6 mL of 2-MBT matrix solution was uniformly sprayed onto the surface of the wood tissue by ImagePrep electronic matrix sprayer in two times (3 mL each time). After 2-3 seconds of spray each time, the slices were then slightly dried for 60-120 seconds. This process was repeated 50 times. The whole spraying process took about 40 minutes. After being sprayed, the wood slices were put into a vacuum tank for 10 minutes drying. During spraying, be sure to keep the spraying environment wet enough. The coated glass slides were installed on the imaging target plate, and then put it into the mass spectrometer.

### MALDI-TOF-MSI

All of the profiling and imaging experiments were carried out using a Bruker Autoflex Speed MALDI-time-of-flight (TOF)/TOF mass spectrometer (Bruker Daltonics, Germany), equipped with a 2000 Hz solid-state, Smartbeam Nd: YAG laser (355 nm). Sample was analyzed in the positive-ion and reflection mode with broadband detection. A mass range of m/z 400 to 1300 were performed. 50 μm laser raster step-sizes was used for the *in situ* detection of low-molecular weight compounds in wood tissue sections. When collecting profiling data, evenly print 30 points each time, 100 shots for each point (if the ideal peak cannot be found, then evenly print 30 points each time, 200 shots for each point). A total of 3000 shots are stacked as one time, and each wood slice was printed 3 times, and 3 profiling data were saved respectively. All wood samples were tested under the same conditions (including laser energy, detector gain, number of shots, etc.).

### Data analysis

For data collected from MALDI-TOF-MSI, the Bruker’s FlexAnalysis 3.4 software was performed for the analysis of the preliminary mass spectra. According to the botanical structure characteristics of National standard of the People’s Republic of China: Hongmu (General Administration of Quality Supervision, Inspection and Quarantine of the People's Republic of China, 2017), the fiber, vessel, axial parenchyma, wood ray and other tissues in the measurement area of each sample were defined. In each sample, these four tissue regions of wood structure were also areas of interest for the distribution analysis of metabolic compounds. The Bruker FlexImaging 4.1 software was used for the reconstruction of the ion map of low molecular weight compounds.

## Results and discussion

### MALDI-TOF-MS analysis of *P. santalinus* and *P. tinctorius*

**Figure 2:**
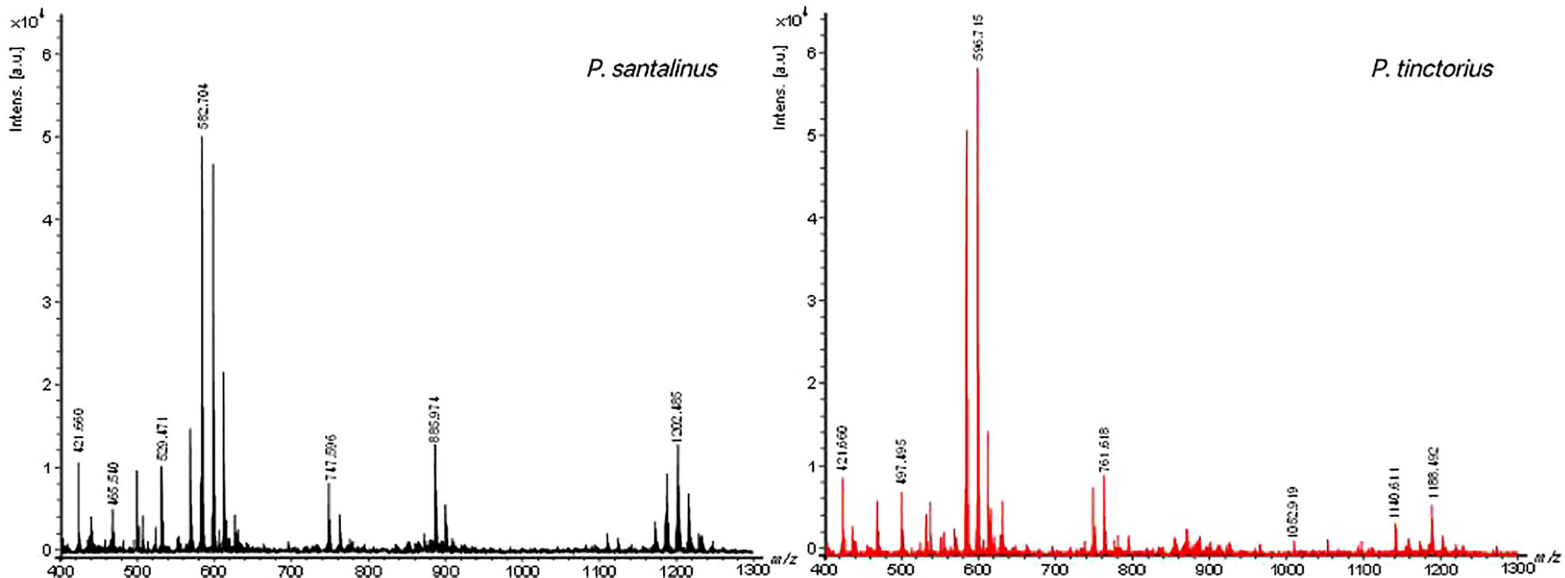
MALDI-TOF mass spectra of wood slices of *P. santalinus* (black) and *P. tinctorius* (red).

The representative heartwood samples were selected from the herbarium, and the chemical fingerprints of two species have been obtained through the detection of MALDI-FOF-MS. The flow chart of analytical methods was shown in **Figure S1**. The overall average mass spectra of *P. santalinus* and *P. tinctorius* gained by MALDI-TOF-MSI under positive ionization mode were showed in **Figure 2**. In the range of 400-1300 m/z, chemical signals were detected in the mass spectra of *P. santalinus* and *P. tinctorius*, and the *P. santalinus* samples showed more high abundant ions than *P. tinctorius.* However, when the wood chip samples of *P. santalinus* and *P. tinctorius* detected by DART-FTICR-MS, the peak range was 200-800 m/z (Zhang et al., 2019a). There was a significant difference between the number of peaks detected in the mass spectra of *P. santalinus* and *P. tinctorius* (**Figure S2**). 202 peaks were detected in *P. santalinus* and 123 peaks were detected in *P. tinctorius* (Table S1). The *P. santalinus* sample has about 64% more peaks than *P. tinctorius.* The wavelength and shape of peaks in the spectra of *P. santalinus* and *P. tinctorius* were very similar. There were 96 common peaks, such as 421.66, 465.62, 497.58, 582.76, 596.77, 747.66 and 886.04 m/z, but the abundance of ions were more or less different. Several significantly different peaks can be observed from the spectra, such as 465.54, 529.47, 885.97 and 1202.48 m/z, which hardly existed in the spectra of *P. tinctorius*, and 497.49 and 1052.91 m/z, which could be found in the spectra of *P. santalinus.* These peaks with significant differences are benefit the differentiation of the two wood species.

### MALDI-TOF-MSI analysis of *P. santalinus* and *P. tinctorius*

**Figure 3:**
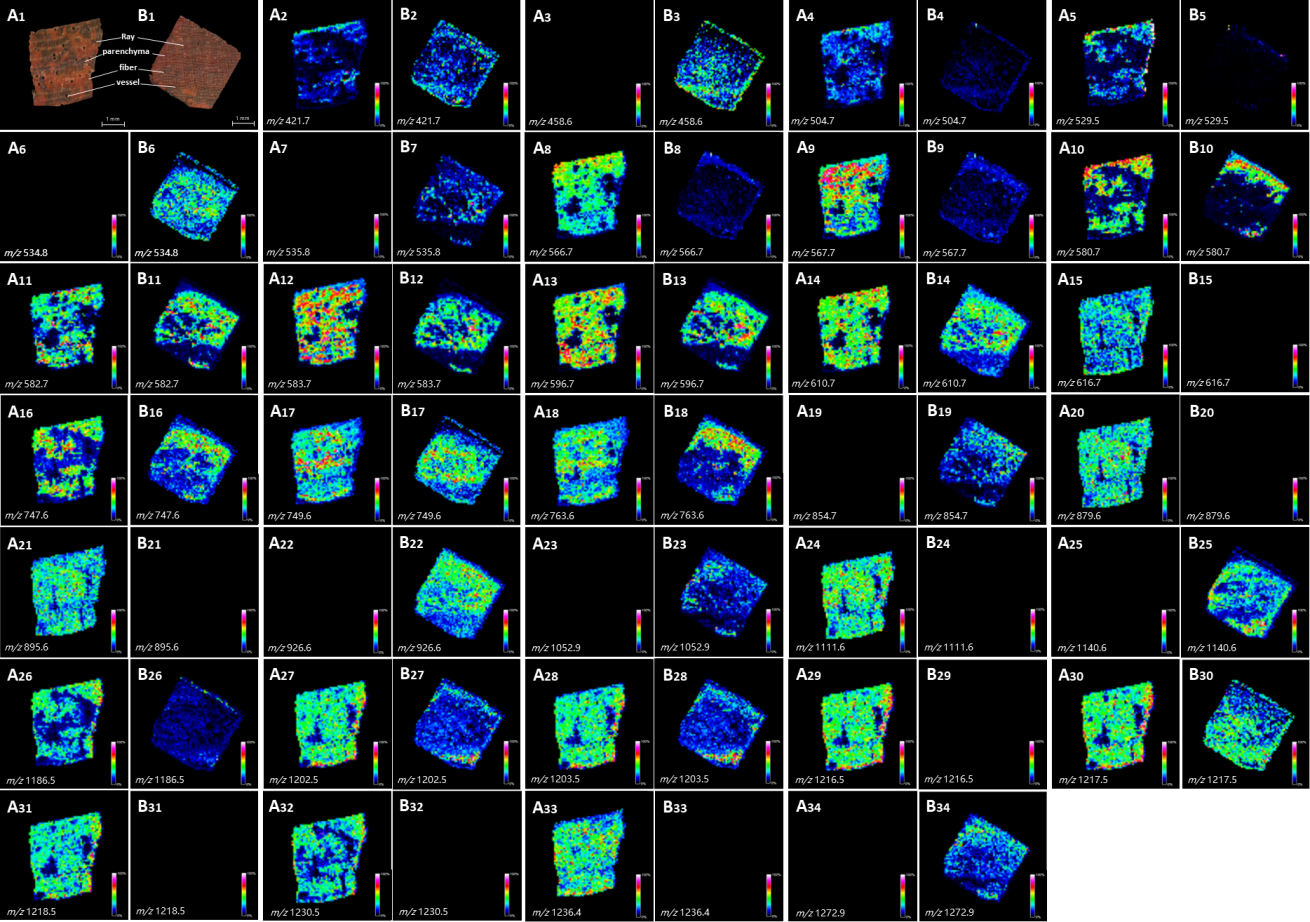
Optical images of tissue sections and localization modes of metabolite ions in wood slices of *P. santalinus* and *P. tinctorius* with significant differences. **(A1, B1)**, optical images of tissue section of *P. santalinus* and *P. tinctorius*, respectively, including four main types of tissue cells. **(A2–A34**, **B2–B34)**, localization modes of metabolite ions in wood slices of *P. santalinus* and *P. tinctorius*, respectively.

**Figure 4:**
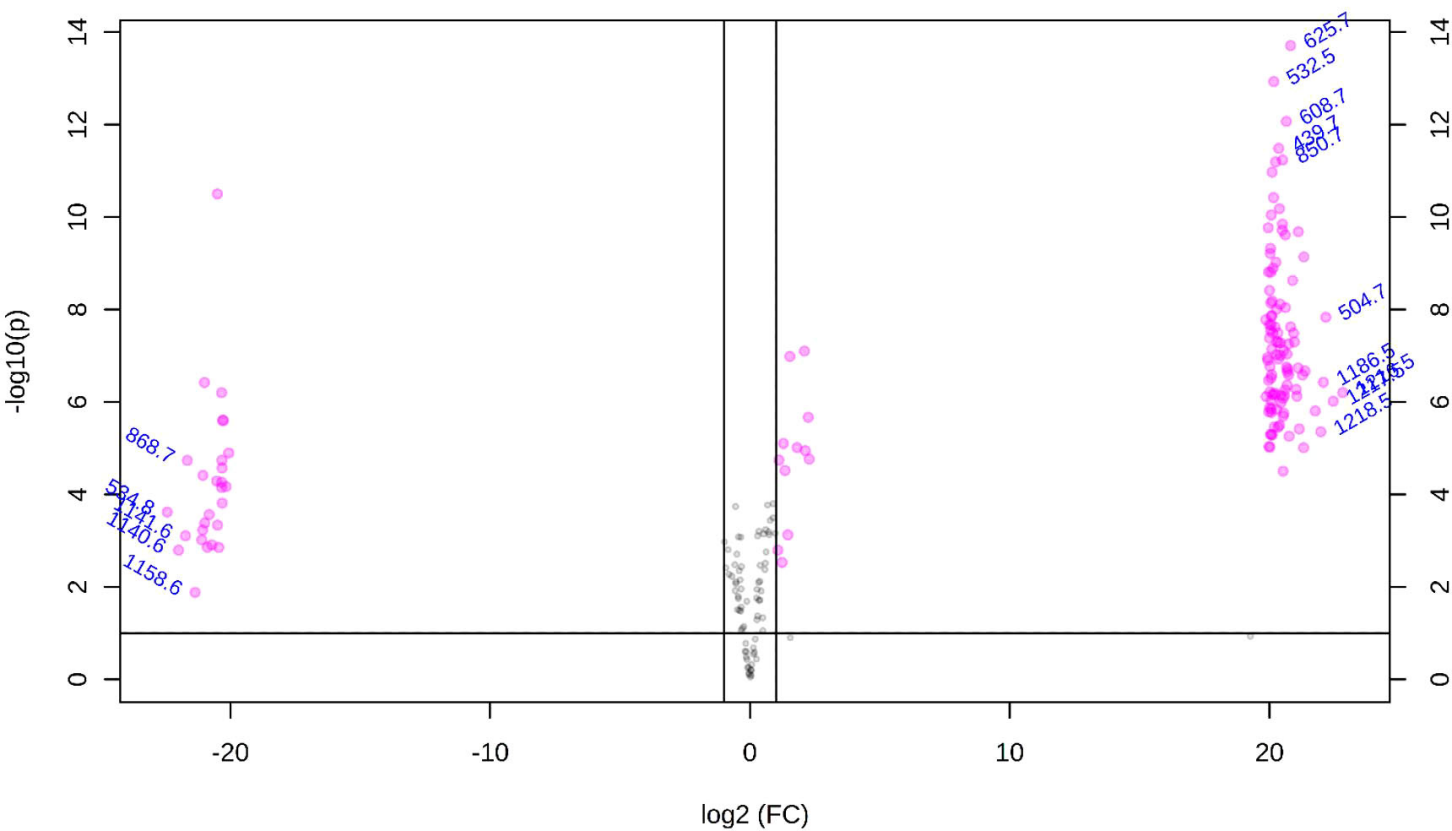
Volcano plot results of *P. santalinus* and *P. tinctorius*.

**Figure 3** showed the tissue heterogeneity distribution of some representative molecules. Ion images directly showed the existence of significantly different metabolites in the two kinds of wood slices. Such as m/z 504.7, 529.5, 616.7, 879.6, 895.6, 111.6, 1216.5, 1218.5, 1230.5, 1236.4 (**Figure 3B5, B15, B20, B21, B24, B29, B31, B32, B33**), among these peaks, the ion abundance of *P. tinctorius* was very small or absent, while at the m/z 458.6, 534.8, 582.7, 854.7, 926.6, 1052.9, 1140.6, 1272.9 (**Figure 3A3, A6, A11, A19, A22, A23, A25, A34**) etc., the ion abundance of *P. santalinus* was very small or absent. This result is consistent with the specific peaks obtained from the mass spectrum, showed in Table S1. Ion image showed that some metabolites with low relative content (in blue) can also be observed and detected by MALDI-TOF-MSI. Common LC-MS will homogenize plant tissue, and metabolites with low content cannot be detected, but MALDI-TOF-MS can detect metabolites with low content at a specific location. MALDI-TOF-MS imaging makes it easy to intuitively lock the category of differential compounds of two wood species, and facilitate further exploration through data analysis. As showed in **Figure 4**, volcano plot displayed two important indicators (Fold change/p-Value). With more than 1.5 times of change and P<0.05 as the screening condition, the differential metabolites that can serve as marker compounds between the two wood species can be screened intuitively and reasonably. The differential metabolites screened from the volcano plot include all the significant differential metabolites shown in the ion image. It fully shows that mass spectrometry imaging has the ability to screen differential expression factors among samples, and is more intuitive than the results of mathematical statistics.

The ion images showed the heterogeneous distribution and relative abundance of different metabolites in wood xylem tissues, which were obviously related to the botanical structure of *P. santalinus* and *P. tinctorius*. It mainly involves four types of tissues, namely fiber, axial parenchyma cell, wood ray and vessel (**Figure 3A1, B1**). The location of metabolites in wood tissue structure can be preliminarily determined by corresponding optical images of tissue section. The anatomical characteristics of the parenchyma cells of *P. santalinus and P. tinctorius* are tangential banded, aliform or confluent, with obvious structural characteristics, so it is better to locate and compare the distribution of metabolites in the ion images. Some metabolites are obviously distributed in parenchyma cells, such as m/z 566.7, 596.7, 610.7, 747.6 and 749.6, the relative content of metabolites in the parenchyma cells of *P. santalinus* was about 50-75%. While in the parenchyma cells of *P. tinctorius*, the relative content of metabolites m/z 610.7, 747.6 and 749.6 can also reach about 50%. Wood fiber is the cell type with the largest tissue proportion in the *Pterocarpus* species. The high abundant metabolites are distributed in the wood fiber cells of the two species, with the widest relative content range. In *P. santalinus*, m/z 567.7, 583.7, 596.7, 610.7, 763.6, 879.6, 1111.6, 1202.5, 1203.5, 1217.5 and 1236.4 etc. were involved, and m/z 596.7, 763.6, 854.7 and 926.6 etc. were involved in *P. tinctorius*. There were also many metabolites with high content in the wood ray cells. Because both *P. santalinus* and *P. tinctorius* are all rays storied, and the rays are thin and densely arranged, the ion image on the wood transverse section showed a clear distribution.

**Figure 5:**
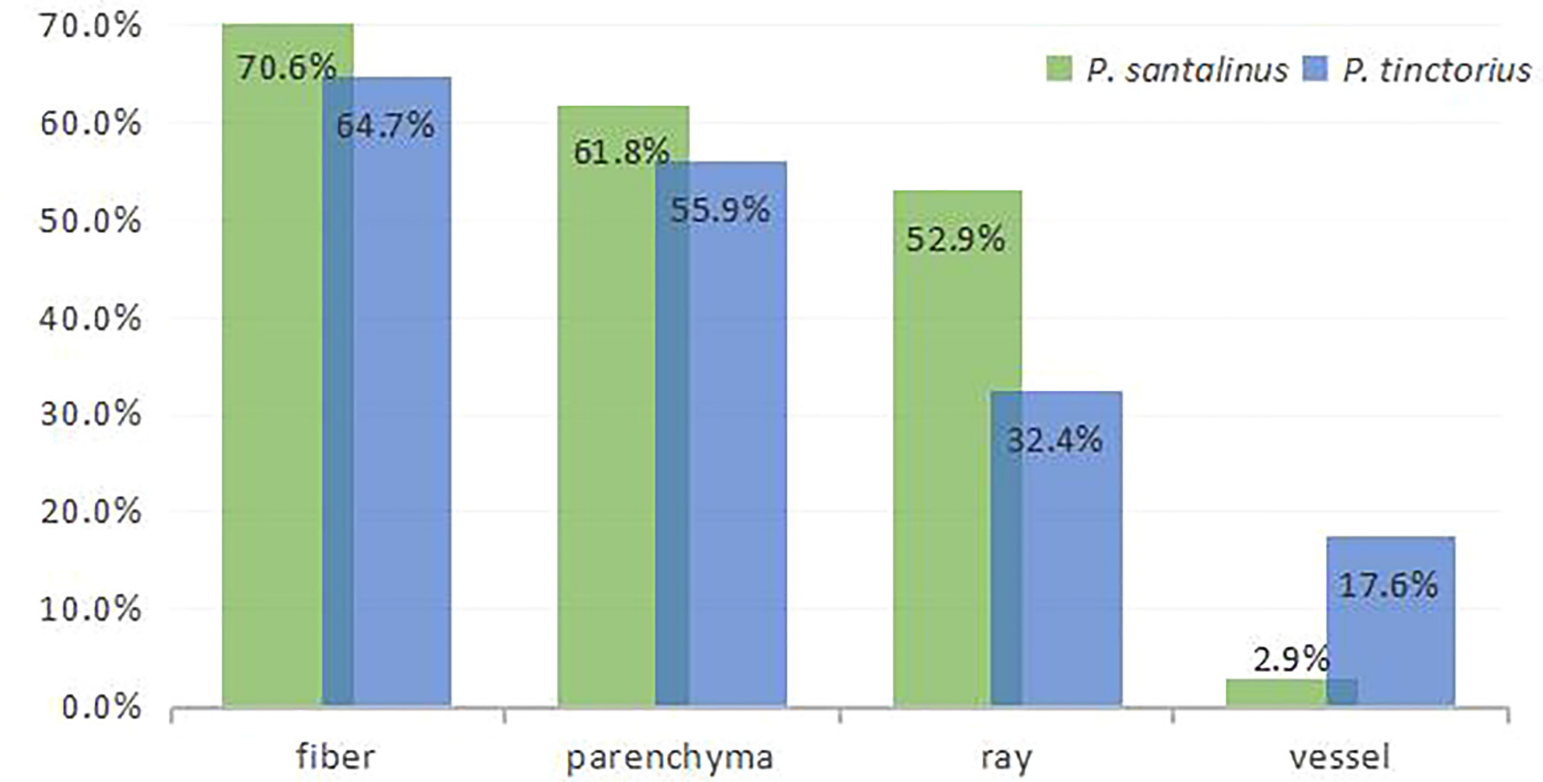
Histogram of tissue distribution of metabolites detected by MALDI-TOF-MSI.

The distribution quantity of metabolites in *P. santalinus* is obviously more than that in *P. tinctorius* (**Figure 5**). The ion image showed that there were few high abundance metabolites in the vessel, which may also be related to the fine wood structure of the two species, namely, the vessel diameter was small, the vessel arranged not too tightly (vessels exclusively solitary or few in radial multiples of 2-3 cells, scattered in 2-5 cells/mm^2^) and the distribution density was also small, so the vessel tissue structure was not fully displayed. In general, there were more metabolites in fibers, parenchyma cells and rays, and less metabolites in vessel were observed by ion imaging, which has a certain relationship with tissue ratio and resolution of ion imaging. The localization of all metabolites revealed by ion imaging combined the chemical information and histological characteristics of plants, which is helpful to understand the coevolution and provenance relationship between two species of *Pterocarpus* wood. MALDI-TOF-MSI analysis made up the gap between morphology and chemistry in complex biological samples.

### Markers for the identification of *P. santalinus* and *P. tinctorius*

PCA (Principal Component Analysis) is a commonly used multivariate statistical method to investigate the correlation between multiple variables. Through linear dimensionality reduction of data, it can generate features that are easy to be found and more easily understood by humans (Rasmus and Age 2014; Hur et al., 2010). The PCA score plot of the MALDI-TOF-MS spectra of *P. santalinus* and *P. tinctorius* were shown in **Figure S3**. The results showed clustering behavior related to the wood species. All samples were divided into two independent groups. The separation trend between the two groups was good, and there was no abnormal point. PCA can effectively process high-dimensional data, but is not sensitive to variables with small correlation. PLS-DA (Partial least squares Discriminant Analysis) integrates multiple linear regression analysis, canonical correlation analysis and principal factor analysis, which can avoid potential problems such as data non normal distribution, factor indeterminacy and model failure to identify. Variable importance on projection (VIP) is the variable weight value of the PLS-DA model variable, which is used to measure the influence intensity and interpretation ability of the accumulation difference of various metabolites on the classification and discrimination of each group of samples. VIP value larger than 1 is a common screening standard for different metabolites (Ahn et al., 2020). The variables are sorted by combining the VIP value and the p value in the t-test, and the selected highly correlated variables can be considered as candidate variables for species differences (Rajalahti et al., 2009). Due to the high dimensional and massive characteristics of mass spectrometry data, the principle of VIP value larger than 1.5 and P value less than 0.05 was used to screen potential markers of interspecific differences by combining single-dimensional and multi-dimensional statistical analysis methods. Using this screening principle, fifteen variables were considered as markers (**Figure S4** and **Table 1**). The fifteen variables were all in the significant screening results of interspecific differences in ion images obtained by MALDI-TOF-MSI (**Figure 3**). Each variable has high VIP value and strong species difference expression. The results of heat map (**Figure S5**) and correlation analysis (**FigureS6**) also confirm this result. It is indicated that the fifteen variables can be used as chemical markers for samples classification of *P. santalinus* and *P. tinctorius*, which can effectively reduce the time and workload of locating compounds and massive data evaluation.

**Table 1:** Potential chemical markers for the interspecific differentiation of *P. santalinus* and *P. tinctorius*.

Mass measured	PLS-DA VIP scores	Relative peak intensity		Localization	
		*P. santalinus*	*P. tinctorius*	*P. santalinus*	*P. tinctorius*
566.7	5.00	30%	5.8%	fiber, pyrenchyma cell	fiber, pyrenchyma cell
1202.5	4.50	26.3%	4.3%	fiber, pyrenchyma cell, ray	fiber, pyrenchyma cell
610.7	3.75	43.7%	25.6%	fiber, pyrenchyma cell, ray	fiber, pyrenchyma cell, ray
1216.5	3.25	14.7%	0	fiber, pyrenchyma cell, ray	—
1203.5	2.75	16.5%	3.4%	fiber, pyrenchyma cell, ray	fiber, pyrenchyma cell
1217.5	2.55	11.5%	0	fiber, pyrenchyma cell, ray	—
529.5	2.50	21.2%	0	fiber	—
534.8	2.45	0	11.2%	—	fiber, vessel
504.7	2.10	9.4%	0	fiber	—
582.7	2.05	100%	87.2%	—	fiber, pyrenchyma cell, ray
1186.5	1.95	8.7%	1.2%	fiber	fiber
567.7	1.80	11.8%	3.1%	fiber, ray	fiber
1140.6	1.75	0	10.7%	—	fiber, pyrenchyma cell
1218.5	1.70	8.4%	0	fiber, pyrenchyma cell, ray	—
580.7	1.65	17.2%	8.6%	fiber, pyrenchyma cell, ray	fiber, pyrenchyma cell

For dry wood, especially the identification of chemical components of wood specimens that have been dried and preserved for many years or even decades, it has always been a difficult point in the field of wood identification, whether it is the use of DNA extraction technology, or the use of chemical component chromatography/mass spectrometry technology for species level identification (Jiao et al., 2018b; Zhang et al., 2019b). Compared with fresh plant materials, such as leaves, roots, stems, or xylem of fresh wood, secondary mass spectrometry can be obtained to identify compounds by MALDI-TOF-MS/MS (Wang et al., 2016; Kuo et al., 2019; Li et al., 2020; Shen et al., 2022). However, for dry wood, compared with fresh tissue, it is difficult to ionize. We have not succeeded in obtaining secondary mass spectrometry using MALDI-TOF-MS/MS in this study. If the secondary mass spectrometry of dried wood samples can be obtained, combined with the ion image of MALDI-TOF-MSI, it can improve the understanding of the unique functions of individual species and their biological functions in plant growth and development, as well as the impact on the macro and micro properties of wood.

MALDI-TOF-MSI is a powerful, cost-effective, fast and robust species classification technology, which has been successfully applied to the classification and identification of microorganisms, herbal active substances and proteins. However, the wood MALDI-TOF-MSI database is not yet available, which does not meet the needs of wood identification and classification. In order to further distinguish species that are closely related and difficult to classify, it is increasingly important to establish, categorize and expand databases.

## Conclusion

Through accurate, rapid and high-throughput MALDI-TOF-MSI analysis of dried wood tissue sections, fifteen potential chemical markers were determined to identify two species of *Pterocarpus.* The results clearly demonstrate that MALDI-TOF-MSI combining multivariate statistical analysis is suitable for the rapid and effective species-level identification of *P. santalinus* and *P. tinctorius.* MALDI Imaging is a powerful tool for visualizing the wood metabolites of *P. santalinus* and *P. tinctorius* for the first time. Some substances with very low content can be detected. Results verified not only the feasibility of using MALDI-TOF-MSI method to identify wood, but also the possibility of using dry wood instead of leaves or fresh tissue as the detection objective, when wood is the plant material to be identified. We hope that in the future, we can carry out chemical fingerprinting research based on MALDI-TOF-MSI for more wood species with similar anatomical characteristics and difficult to distinguish, build a database, and provide technical support for curbing illegal timber logging activities and for protecting endangered and valuable wood species. The compatibility between different mass spectra and the in-depth mining analysis of chemical markers will also be the future research direction of the application of chromatography-mass spectrometry technology in the analysis of different compounds.

## Data availability statement

The raw data supporting the conclusions of this article will be made available by the authors, without undue reservation.

## Author contributions

BLiu, YL and YF contributed to the conception and design of the study, QC, LT and LZ performed the experiment, XZ and BLi performed data analyses, and BLiu and WF wrote the manuscript. All authors contributed to the article and approved the submitted version.
