# Supplementary material for: Screening of potential chemical marker with interspecific differences in Pterocarpus wood and a spatially-resolved approach to visualize the distribution of the characteristic markers
Source: Front Plant Sci. 2023 Feb 14;14:1133848. doi: 10.3389/fpls.2023.1133848 (PMC9971912; doi:10.3389/fpls.2023.1133848)
Supplement: Supplementary file 1 [file DataSheet_1.pdf]

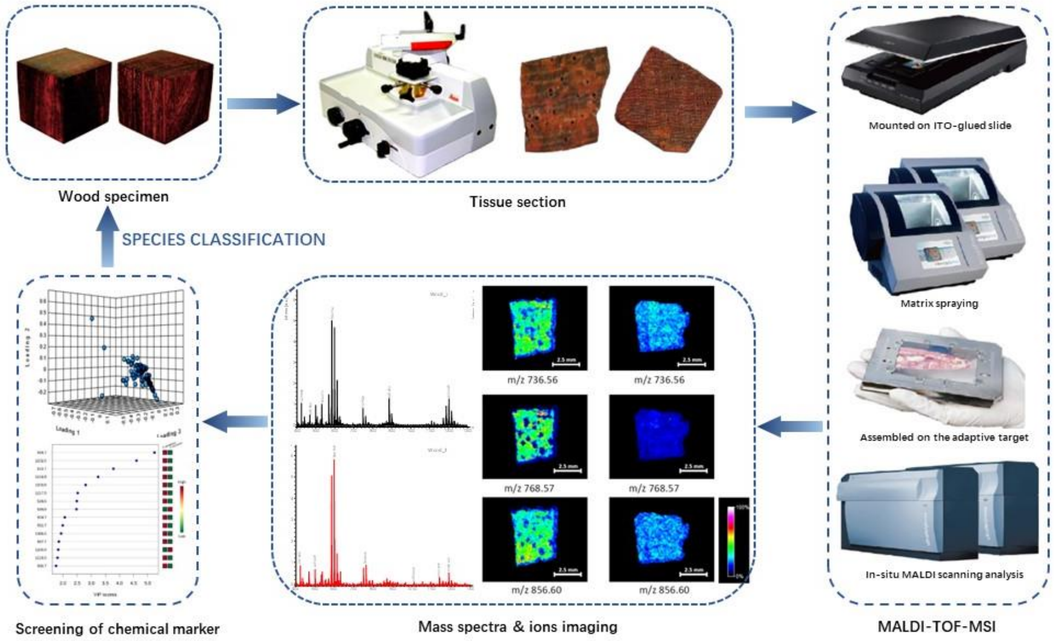

Fig. S1 Flow chart of analytical methods in this study

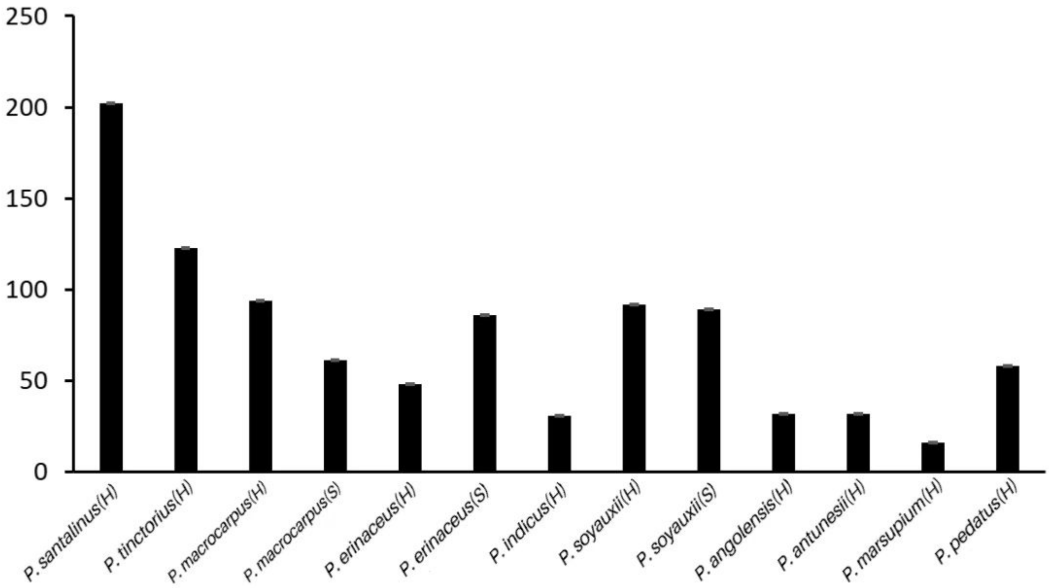

Fig. S2 The number of peaks detected by MALDI-TOF-MSI in 10 *Pterocarpus* species. (H and S) Heartwood and Sapwood.

Tab. S1 The common and unique peaks of *P. santalinus* and *P. tinctorius*

Names	Total	Elements
P. s & *P. t*	96	400.5 419.6 420.7 422.7 423.6 433.6 435.6 437.6 438.6 455.6 465.5 466.5 467.5 469.5 497.5 498.5 499.5 529.5 530.5 531.5 553.7 554.7 561.5 566.7 567.7 568.7 569.7 580.7 582.7 581.7 583.7 584.7 585.7 586.6 594.7 595.7 596.7 597.7 598.5 599.7 600.6 604.7 610.7 611.7 612.7 613.7 614.7 615.7 616.7 618.7 626.7 627.7 628.7 629.7 630.6 634.7 642.7 662.4 762.6 670.7

		694.4 746.6 748.6 747.6 749.6 761.6 763.6 764.6 775.6 779.6 793.5 852.7 866.7 867.7 876.7 880.6 882.6 910.6 920.6 924.6 1142.5 1173.5 1172.5 1174.5 1187.5 1188.5 1189.5 1190.5 1191.5 1202.5 1203.5 1204.5 1220.4 1221.5
P. s	106	402.5 404.7 405.7 406.5 424.5 434.6 439.7 440.5 442.5 444.7 447.6 448.7 451.6 460.6 462.6 463.5 464.6 465.5 472.5 479.6 504.7 505.7 506.7 511.5 519.5 520.5 522.7 528.5 532.5 536.7 538.6 550.7 555.5 563.5 564.7 565.7 578.7 579.7 605.7 608.7 609.7 622.7 625.7 632.5 634.5 640.7 719.7 731.6 732.6 733.6 745.6 750.6 758.7 760.7 772.7 774.7 776.6 777.6 834.7 836.7 848.6 850.7 862.6 864.6 878.6 879.6 890.6 892.6 894.6 895.6 896.6 908.6 909.6 1082.6 1094.5 1110.5 1111.6 1112.6 1124.5 1126.5 1157.5 1170.5 1171.5 1175.5 1176.5 1185.5 1186.5 1200.5 1201.5 1205.5 1206.5 1214.5 1215.5 1216.5 1217.5 1218.5 1219.5 1125.5 1230.5 1231.5 1232.4 1234.4 1235.4 1236.4 1248.5
P. t	27	401.6 453.6 458.6 468.6 534.8 535.8 548.8 600.6 646.6 780.6 794.6 795.6 854.7 868.7 869.7 870.7 926.6 964.9 1008.9 1052.9 1140.6 1141.6 1154.6 1158.6 1159.6 1228.9 1233.5 1272.9

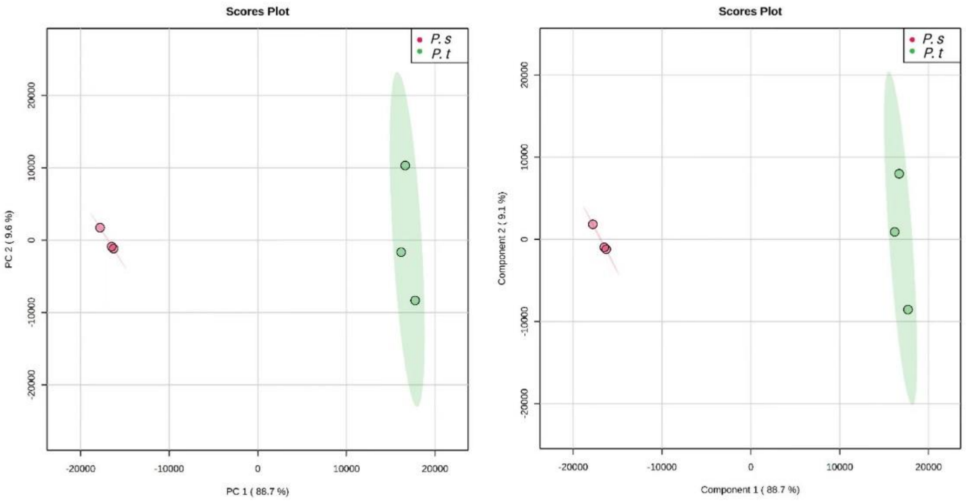

Fig. S3 Score Plot of PCA of the MALDI-TOF-MS spectra of *P. santalinus* and *P. tinctorius*

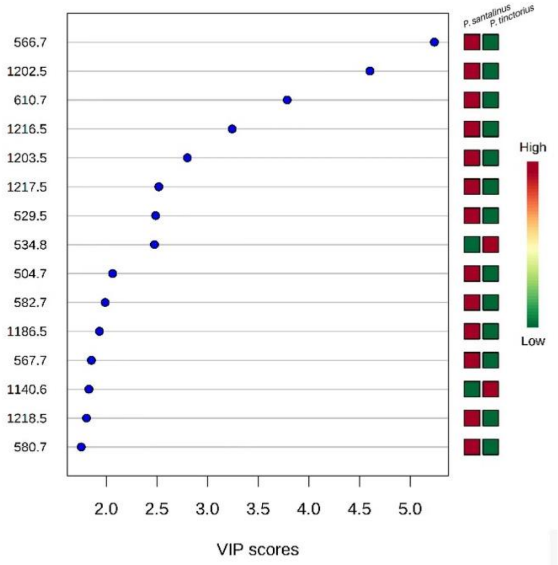

Fig. S4 VIP scores of PLS-DA

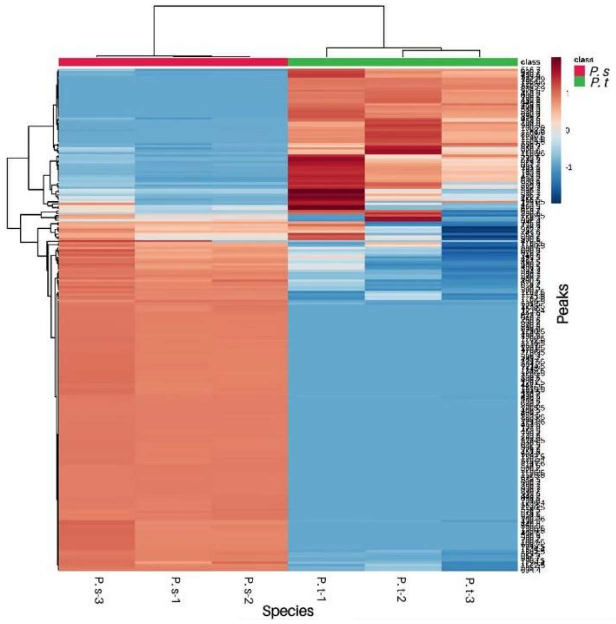

Fig. S5 Heatmap and HCA results based on MALDI-TOF-MS

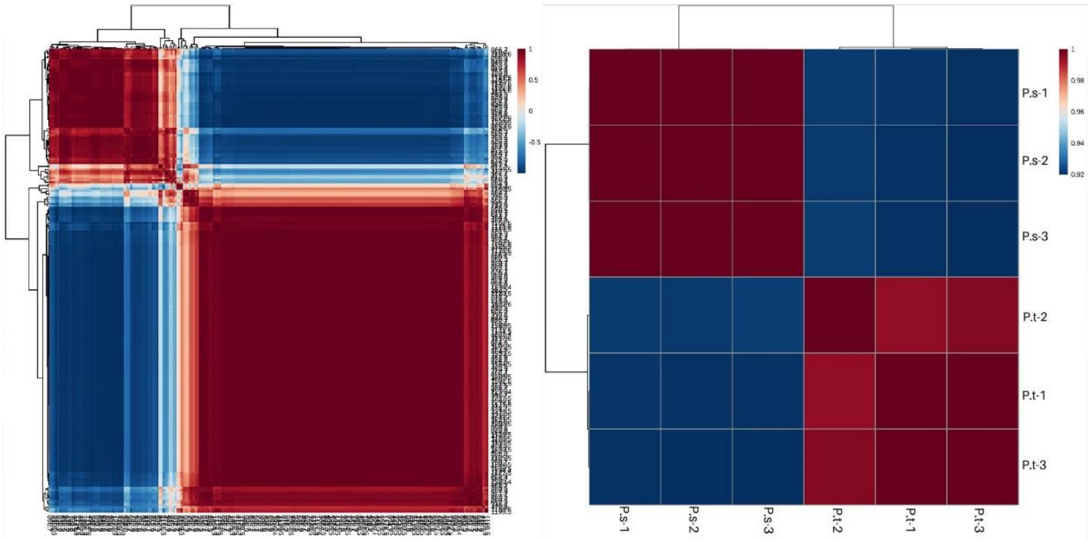

Fig. S6 Correlations Analysis results based on MALDI-TOF-MS
